# Progress of Veterinary Science

**Published:** 1882-01

**Authors:** 


### PROGRESS OF VETERINARY SCIENCE.

---

Rabies, a Possible Cause and Probable Preventive.—This "possible cause," according to Dr. L. L. Dorr, lies in the mixing of breeds. Hydrophobia may exist in all dogs, but it never originates in any but mongrels. The destruction of mongrels might stamp out the disease, only, unfortunately, no one knows exactly what dogs are of pure breed and what not. The "probable preventive" is a visit of the bitten person to the Pacific coast. No person has ever died of hydrophobia in that region, so far as is known.—*Medical Record.*

Precautions against Hydrophobia.—The Prefecture of Police have caused to be placarded throughout Paris the following precautions, to be observed in the case of persons bitten by a dog supposed to be mad. They are the outcome of a special hygienic commission composed of the following gentlemen: MM. Chatin (President), Gaubaur, Léan Colin, Trélat and Dujardin-Beaumetz:

1st. The bitten part should be made to bleed as freely as possible. It should then be washed with water—a jet being preferably used if near by. If water be not at hand, any other harmless liquid may be substituted.

2d. Cauterization should now be resorted to, and among the best caustics are the sollowing: Vienna paste, butter of antimony, zinc chloride and iron perchloride.

3d. The success of cauterization entirely depends upon the promptness with which it is done; therefore it should be resorted to immediately after the bath, by a layman, if a doctor be not present.

4th. Cauterization by ammonia and the various alcohols are proven to be qnite inefficacious.

Antidote for Hog Cholera.—I will say that I have been manufacturing molasses for the past twenty years, and have never known of a hog dying with cholera while being fed on the waste from a sorghum factory, but have known cases where hogs, after beginning to take the disease, and after some of them had died, the remainder—though some of them sick—were entirely freed from the disease by eating the waste. My neighbors frequently haul away the waste and use it, and it has in every case had the same effect, not a hog dying after its use.—*Indiana Farmer.*

Food for Carrier Pigeons.—A distinguished Belgian pigeon fancier, whose birds have carried off some of the best prizes to be had, including the famous two thousand francs prize for a race between Rome and Brussels, gives the following as the best food for carrier pigeons. Some of the birds bred by this fancier are worth a thousand francs apiece: Wheat, maize, beans, dried peas, a little millet seed and some buckwheat, together with some old plaster mixed with salt. A change of food is as desirable for birds and animals as human beings; it is well to ring the changes upon the above-named edibles.

A Remarkable Mode of Zoölogical Distribution.—M. Méguin lately presented to the Société de Biologie a collection of fifteen leeches, of a species foreign to those found in France. They were adhering to the walls of the mouths of horses just returned from the Tunis campaign. They had been camping near Bizerte. All the brooks of Northern Africa contain that kind of leeches, called *hæmopis sanguisuga*, whose jaws are too weak to bite the skin, so that they enter into the animals' mouths when these come to drink, and cling to the mucous membrane. They may live in this situation for weeks, causing hæmorrhages, and even asphyxia, if they enter the larynx. But they die almost immediately when introduced into the stomach or rectum.—*Chicago Medical Journal and Examiner.*

The Effect of Long-continued Minute Doses of Mercury on Animals.—Schlesinger, in a prize essay, published in full in the *Archiv für Exp. Path.* (Bd. xiii., S. 352), gives the following conclusions:

Rabbits, and particularly dogs, bear without harm the continuance for a year of small doses of chloride of mercury and sodium. Compared with controlled animals, however, they show a certain increase in weight, while examination shows a surprising increase in the number of red blood copuscles. In spite of this, the urine of these animals shows no change in its ordinary constituents, and no sugar or albumen present at any time.

The animals killed after taking mercury during a shorter or longer period show no disease of any organ. They only show a large accumulation of fat n those localities where fat is usually found in dogs.

Flesh and Fat Producers.—The *American Agriculturist* makes up, from the published analyses of the most eminent agricultural chemists, the following table exhibiting the relative nutritive value of different feeds. It is said to correspond strictly with the experience of many noted English feeders:

| | Flesh. | Fat. |
|---|---|---|
| Turnips | 1 | 5 |
| Rutabagas | 1 | 7 |
| Carrots | 1 | 7 |
| Mangels | 2 | 8 |
| Straw | 3 | 16 |
| Potatoes | 2 | 17 |
| Wheat and barley | 12 | 67 |
| Linseed | 23 | 92 |
| Hay (early cut) | 8 | 50 |
| Buckwheat | 9 | 60 |
| Rye | 11 | 72 |
| Oats | 12 | 63 |
| Corn | 12 | 68 |
| Linseed cake | 28 | 56 |
| Bran and coarse mill stuff | 31 | 54 |
| Decorticated cottonseed cake | 41 | 77 |

It will be seen from the above that cottonseed meal has no superior as a flesh-former, and that for fattening it is better than every other article of stock feed. In a very short time it has established itself, both in this country and in Europe, as the food for beef cattle and for dairy purposes.

Pasteur's Vaccinations.—Statistics brought up to Oct. 1, show that the inoculations of splenic fever, according to Pasteur's method, were performed on 160 flocks, comprising 68,900 sheep, of which 33,576 were vaccinated and 21,938 left uninoculated, so as to judge of the results of the difference of treatment. Before vaccination, the losses caused by splenic fever amounted in the whole of the flocks, to 2,986 animals. During vaccination, and until its effects were perfected, 260 sheep out of the whole number of 33,576 perished. During the same period, the mortality rose to

366 out of the group of 21,938 which were not vaccinated. When the effects of vaccination were complete in the first group, the mortality from splenic fever fell to 5. This rate has persisted up to the present time; and the next statistical account will give, it is expected, the same satisfactory results as regards the groups of animals vaccinated and left unvaccinated.

A New Cure for Snake-Bites.—All who are or have been in danger from venomous snakes will be glad to hear of a new cure, which seems capable of very rapidly destroying the poison. An account of experiments by M. de Lacerda has recently been presented to the French Academy of Science by M. de Quatrefages, in which permanganate of potash has been hit upon and found perfectly successful as an antidote to serpent poison (derived from the Brazilian Bothrops), whether injected beneath the skin or into the veins of dogs. The solution of permanganate of potash, diluted to the extent of one to one hundred with distilled water, was injected, in one series of experiments, within a minute or two of the injection of the poison, and in no case did the ordinary consequences of the poison appear; while the venom at the same time was proved to be fully venomous by its action on other dogs without the administration of the antidote. In other cases, where the poison was injected into the veins of dogs, followed in half a minute by the antidote, its consequences were arrested; while a still more striking result was the cure of dogs when already very seriously suffering from the effects of the poison. In two or three minutes the worst consequences disappeared under the action of the permanganate, and the state of general prostration was recovered from in at most five-and-twenty minutes.—*Land and Water.*

Controlling Sex.—To those interested in knowing how to control the sex of offspring of animals, the following statement from Dr. George Watt, in the *National Live-Stock Journal*, may be of some use. These observations were carefully and faithfully made, and extended over a period of nearly fifty years. His conclusions are: "When first developed so as to be viable, the ovum of viviparous animals is male, and if vivified at this stage, the offspring is male; and at some period in its progressive development, before its viability is lost, it becomes female, and from conception at this stage the offspring is female. This is in accordance with 'Adam was first formed, then Eve;" and may it not be that creation is the precursor and type of procreation? At least we have many hints of harmony between the two. If this theory is true, when we wish to breed a male, the female should be mated at the earliest hour she will tolerate copulation. If female offspring is desired, mate as nearly as practicable to the close of the "heat" period.

A Breeding Mare Mule.—A breeding mare mule was recently exhibited at the Jardin d'Acclimatation in Paris, which has produced three colts. As the French *savants* have hitherto been very incredulous as to reports of mule breeding, it is stated that they carefully inquired into this case, and became satisfied that it was true. We have heard of mare mules occasionally breeding in America, but we do not recollect the year and locality of this, or whether the sire was a jack or a horse, and shall be obliged to any of our readers who can furnish these particulars; also, what sort of an animal the produce turned out to be. In the above instance of mule breeding in France the sire was a horse.

Protection of Cattle against Charbon.—M. Pasteur has addressed he following letter to the Prefect of the Seine et Marne, on the subject of the numerous demands addressed to him by various veterinary surgeons, through the medium of the Couseil-Général, on the subject of the supply of the new vaccinal matter which he has introduced for the purpose of preventing black water or charbon. The demand for the vaccinal matter, it seems, now already far exceeds the available supply: "Monsieur le Préfet. —I was in London when you came to Paris to explain to me your desire of calling upon the Conseil-Général of Seine et Marne to set aside a grant for creating a small laboratory for furnishing my vaccinal matter by which to protect animals against charbon. I think that such a grant will be premature. Many questions of detail still remain to be solved, which can at present only be solved by me. For example, all the vaccine which has passed out of my laboratory for the last month has been obtained by recent culture. An arrangement such as you propose implies storing, and the employment of tubes prepared at long intervals. Difficulties which will, no doubt, be easy to remove by further research at present occur to impede the preservation of this vaccinal matter in tubes. Do not, however, fear a famine of this precious liquid for next year. This pest does not prevail to any considerable extent during the winter, and at present I already possess the means of making it on a large scale. I shall find, after the holidays are over, one or two hectolitres of culture fluids already prepared; and by the month of March or April, when vaccination may be most usefully commenced, there will be, I hope, vaccine prepared for a million of animals. A very intelligent person in my laboratory will have no other occupation during the whole of the year than the preparation of this vaccine, and will have an assistant. There will be a depot of vaccinal tubes here; and I shall hope to be able to place it in the hands of veterinarians at five centimes per beast, which is about what it costs. During the first year it will be necessary to watch the state of the preserved vaccine and the permanence of its protective powers. A provincial establishment would occupy me and take up my time as much as the supply laboratory which I shall arrange here. When the regularity of working of that which I am here arranging has been proved it will be time to follow out our plan, and you can then count on my readiness to serve you. I have the satisfaction to inform you that, thanks to the devotion of all assistant fellow-workers and laboratory servants, the number of animals already vaccinated reaches nearly 30,000 sheep and some hundreds of oxen, cows and horses."

Hydrophobia and its Cure.—Among the many advances which the germ theory of disease is now making, few could be more interesting than that which would enable us to cope with the terrible malady, hydrophobia, justly dreaded from its fatality and nature; and already we are beginning to get a glimpse of a possible cure for the disease.

M. Pasteur, the pioneer and leader of this branch of practical medicine, found that it was possible to "cultivate" organism from the saliva of a boy who had died of hydrophobia, and that after this artificial cultivation, the organism injected into a rabbit produced a disease which, though rapidly fatal, differed in its symptoms from those of true hydrophobia. M. Galtier, taking up the inquiry and acting on a principle which has been found to hold good in another disease, viz., that the effects of a virus differ very considerably according as it is introduced into the veins, and thus into the circulation directly, or into the tissues under the skin, and so indirectly into the circulation, injected some hydrophobia virus into the veins of a number of sheep and goats. The effect was no inconvenience to, or symptoms of disease in, the animals, and further it was found that they were now rendered protected against the action of hydrophobic poison when received into the body in the ordinary way, while other sheep and goats not protected in like manner, quickly succumbed. Further experiments are now being made by the same French veterinary surgeon to ascertain if this injection of virus into the veins has any effect on the development of the true hydrophobic poison communicated by the bites of animals. As is well known, the laten period of this disease—that is, the time which elapses from the reception of the poison till the manifestation of the symptoms—is considerable, and it has been conceived possible that the injection of hydrophobic virus into the vein, after it has been communicated by the bite of an animal, may produce a mild form of the disease, which will render the body, in a way that we cannot explain, incapable of serving as the place of the development of the true disease, and thus establish a cure. Whether experiments will confirm this or not it is premature to judge, but it may be added that in the case of small-pox, in cases where the disease has been already contracted, vaccination has no effect on it.

There is another remedy, which has of late years been tried for hydrophobia, and it is said in Germany with success—that is, the arrow poison of the South American Indians, known as urari, curari, or woorali. This substance has the effect of preventing motion by paralyzing the nerves which go to the muscles known as motor nerves, and as one of the worst symptoms of hydrophobia is interference with respiration by the respiratory muscles being spasmodically contracted, it seemed likely that this substance, by rendering the nerve less capable of conducting the impulses which placed the muscle in contraction, might be of use, and there are several cases in which this drug has been accredited with effecting a cure.

The Stomachs of the Horse, Cow, Sheep and Pig.—A. Stutzer (*Jour für Landwirthschaft*) has tested the comparative value of pepsin obtained from the stomachs of the above animals, and found that that from the pig is the most active.

Rapidity of Stomach Absorption in the Dog and Cat.—H. Tappeiner (*Zeitschr für Biol*) has determined the rapidity of absorption of various substances in the stomach of carnivora when the pylorus is tied. He fed the animals with definite amounts of food in solution. The result showed that very little absorption would take place under the conditions imposed. Secretion seemed to take place more rapidly than absorption, thus keeping the stomach full of fluid.

The Digestion of Fishes.—Rochet and Mourrut (*Compt. Rend.*, 90, 879–881) have made a number of experiments regarding the stomach digestion of various fishes of the genus *Scyllium* and *Lophia piscatorius*. They found that the activity of the pepsin obtained from gastric mucous membrane differed very much in different fishes. Four grains of the gastric mucous membrane of the scyllium digested six grains of fibrin in an hour. This was eighteen times as strong as a corresponding amount of the mucous membrane from a lophia.

The acidity of the gastric juice of fishes is very great, varying between six to fifteen grams (ʒ iss to ʒ iv) of hydrochloric acid litre (quart). This is somewhat greater than that of the human stomach, which is about one grain per litre.

On the whole, the fish gastric juice seems to resemble that of a dog.

Muscular Strength in the Jaw of the Crocodile and Dog.—MM. P. Regnard and R. Blanchard have made some experiments with crocodiles to determine the power of their masseters.

In one crocodile, ten feet long, weighing 115 pounds, the animal in closing his jaws developed a resistance equal to 1,400 pounds (700 kilos.) at the point of insertion of the masseter.

In a dog weighing 40 pounds a resistance of about 330 pounds was obtained.

The proportional strength of jaw to weight of animals is as follows: 1 pound of crocodile will bite with a power of 12 pounds; 1 pound of dog will bite with a power of 8 pounds.

Dog Disease in the Arctic Regions.—Dr. Colan writes, in the English *Veterinary Journal*, that the dogs in Greenland are subject to a kind of madness much resembling rabies, though differing from it in some marked particulars. It first appeared in 1859, and was supposed to have been communicated to the dogs by the foxes. It was exceedingly contagious between dog and dog, but no human being was affected with true hydrophobia from the bite of the dog. It was so severe that the Danish government had to take decisive measures to stamp it out. When an autopsy was made, no cause for the disease could be found, but there was a pitchy substance in the intestines, and ulceration for four inches on either side of the ileocœcal valve. The Doctor asks, "Is it not possible that the dog disease of the Arctic regions is but ripening rabies, which to the southward of 70° north, shows itself in the rabies, which cause hydrophobia? Or is it thât hydrophobia is modified in passing through the bodies of carnivorous animals going north, such as foxes and wolves, and becomes the disease we see in North Greenland?" It is a well-known fact that when rabies first appeared in some countries it was less virulent than after a number of years; if so, might it not now be developing in Greenland?

Diseases Among Texas Cattle.—Dr. Jos. R. Smith, U.S.A., read a paper upon this subject at the American Public Health Association. He gives a table of the pathological changes observed in cattle which have died from Texas fever. His report concludes with some observations upon the question whether this disease is endemic. He says: "I think the facts here reported prove that the herds of Texas cattle, when grazing on their prairie pastures, are singularly free from disease—certainly from any disease recognizable by the ordinary tests of disease, viz.: symptoms and pathological appearances. If so many assertions had not heretofore been made, and so many witnesses heretofore cited, where Texas cattle, apparently healthy, had infected other cattle, mingling with them, crossing their line of march, or following them in their grazing grounds, the inference from the foregoing would be undoubted, that there was no danger to be apprehended to other cattle by exposure to cattle from Texas. Whether some of these instances do not deserve another interpretation, is in my mind a question. Certainly, in many of them, accounts of which I have perused, there is no such rigorous analysis of facts as shows the conclusions drawn to be inevitable.

From the fact that imported cattle from the North soon after arrival in this Southern clime sicken and die in great numbers, it cannot be logically concluded that such death results from infection from Texas cattle, and certainly in many instances of this kind not the slightest evidence is offered of any intermingling of imported and natives. On the contrary, as in the case quoted on page 244, Transactions American Public Health Association, 6 vol., it is stated by the owner of the Durham stock that sickness and death appeared among his newly imported Durhams before their arrival at and on the way to their home near Mason.

It is well known to importers of fine horses in this country that considerable risk attends their importation, and this when kept by themselves in cities and in private stables. Emigrants, too, aro subject to disease, not all of them, but a sufficient proportion to make the matter an element in considering the subject of emigration.

Now this sickness of newly-arrived men and horses has always been considered due to some acclimating process. The idea does not lack probability that the sickness and mortality among the imported cattle are due to a similar acclimating process.

RELATIVE DISTRIBUTION OF TRICHINÆ SPIRALIS IN THE MUSCLES OF THE HOG.—The following table, furnished by Dr. J. T. Payne in a report on trichinæ in Southern hogs, will be of value to veterinary inspectors. It shows the number of trichinæ found in one grain of each of the following named muscles:

| | Pectoral. | Diaphragm. | Abdominal. | Ham. | Total. |
|---|---|---|---|---|---|
| Hog No. 1 | . | 9 | . | . | 9 |
| Hog No. 2 | 4 | 9 | 2 | 3 | 18 |
| Hog No. 3 | 31 | 74 | 65 | 218 | 388 |
| Hog No. 4 | 262 | 120 | 105 | 188 | 675 |
| Hog No. 5 | . | 20 | 22 | . | 44 |
| Hog No. 6 | 12 | . | 22 | . | 34 |
| Hog No. 7 | 60 | 76 | 152 | 4 | 222 |
| Hog No. 8 | 98 | 52 | 166 | 136 | 442 |
| Hog No. 9 | . | 8 | 3 | . | 11 |
| Hog No. 10 | 181 | 294 | 119 | 92 | 686 |
| Hog No. 11 | 39 | 118 | 79 | 22 | 258 |
| Hog No. 12 | . | 120 | 17 | 260 | 397 |
| Hog No. 13 | 27 | 83 | 13 | 29 | 152 |
| Hog No. 14 | 14 | 64 | 21 | 33 | 132 |
| Hog No. 15 | 19 | 40 | 37 | 16 | 112 |
| Hog No. 16* | . | . | . | . | . |
| Hog No. 17 | 13 | 49 | 25 | 14 | 101 |
| Hog No. 18 | . | 17 | 15 | 3 | 35 |
| Hog No. 19 | 2 | 7 | 19 | 6 | 34 |
| Hog No. 20 | 13 | 22 | 25 | 9 | 69 |
| Hog No. 21 | 16 | 24 | 13 | 38 | 91 |
| Hog No. 22 | 92 | 219 | 85 | 102 | 498 |
| | 885 | 1,815 | 1,003 | 1,173 | 4,386 |

* Not counted, but very highly infected with trichinæ.

From this it appears that the parasite is about equally distributed between the diaphragm, the abdominal muscles and the ham.

EXTRA-UTERINE PREGNANCY IN A QUAIL.—Dr. Lydston, of Chicago, describes the following in the *Medical Record*: "A tumor was discovered in dressing a quail. It was situated in the peritoneal cavity, and was apparently free from adhesions to the intestines and abdominal walls. It measured a fraction less than seven inches in circumference, and weighed two and a quarter ounces. On section, it presented a number of laminæ, composing the walls of a cyst, about as large as a small almond. The two internal layers appear to be distinct from those external to them, and separated from the latter by a layer of grumous material resembling the yolk of an egg after partial cooking. The cyst itself was empty. Microscopically, the laminæ composing the cyst walls presented irregularly inasculating fibres of connective tissue, and the grumous material above-mentioned, contained oil globules in abundance, amorphous and granular matter, and what strongly resembled tesselated epithelial cells, the outlines of which were not all clear. The external layer of the sac was rough and corrugated, and of a brownish color, the remaining layers being greenish white. The tumor was evidently an abortive ovum, which failing to enter the oviduct, and falling into the peritoneal cavity, had become encysted by successive layers of fibrinous deposit. The causes of its failure to enter the oviduct was probably an occlusion of the latter, either from injury or inflammation. It presents a striking analogy to the process of encystment which occurs in extra-uterine pregnancy." Dr. Lydston had never heard of but one similar case, which had occurred in a domestic fowl.

The Temperature of Cattle.—A few words in reference to the temperature observed in cattle:

Before beginning my study on this subject, my ideas of the temperature of cattle were quite vague, and the first reports received from Corpus Christi, where the average temperature appeared as over 103 degrees, staggered me.

Could their natural temperature be so much higher than that of the human body? But not only were the next reports confirmable to the first one, but *all* received were to the same effect, and the average temperature of forty-five cattle, as reported to me by five different observers, was 102.59 degrees.

I know of no observations made for the purpose of determining the normal temperature of these animals.

In looking over the volume of "Researches, Physiological and Anatomical," by John Davy, published in London, 1839, I found some experiments reported by him.

He states that the temperature of the blood of an ox flowing from the carotids was 100 degrees in summer, at Edinburgh, and that the temperature of an ox ascertained the same way in Kandy, May 28, was 102 degrees, the atmosphere at the same time being 80 degrees.

When further I saw the temperature of fever in these animals, as observed by Prof. Gamgee, given as from 106 to 110 degrees, I came to the conclusion that the normal temperature of cattle exceeds the human temperature four or five degrees.—*Dr. J. R. Smith, in Report to American Public Health Association.*

The Size of the Spleen in Cattle.—The question of the size and appearance of the normal splenic spleen in cattle is of great importance, since that organ is so often affected in contagious diseases. Dr. Jos. R. Smith contributes the following facts upon this point. He says:

The mean of all my figures gives the weight of the spleen as exactly 2 lbs. The spleen of Texas cattle, weighed under the supervision of Dr. Rauch, in Chicago, gives an average weight of 2½ lbs., while those of the natives reported by Dr. Rauch (Illinois probably, or vicinity), average less than 1½ lbs. The ages of these cattle are not given.

Dr. Powell, at Fort Griffin, found that the weight of spleens increased regularly at different ages up to five. The figures deduced from all my reports agree with this. The average weight of spleen, classed according to the age of animals, was as follows:

In animals aged two years, 1.68 lbs. In three-year animals, 2.18 lbs. In animals aged 4 years, 2.45 lbs. In five-year animals, 2.85 lbs.

If the animals reported in Chicago were three, four and five years old, according to the weights just given for those ages, the weight of the spleen should have averaged 2½ lbs.

The spleen farther appears, according to my observations, to vary with the weight of the animal generally. From Fort Ringgold, Texas, I have quite full data from seventy-four animals killed for beef, and I take this place because every animal reported from there is fully reported.

| | |
|---|---|
| Of all these beeves the average net weight was . . . . . | 301 lbs. |
| Of 38, the weight of whose spleens was between 1 and 2 lbs., average weight was . . . . . . . . . . | 267 lbs. |
| Of 27, the weight of whose spleens was between 2 and 3 lbs. . | 346 lbs. |
| Of 9, the weight of whose spleens was between—and 3 lbs. . | 312 lbs. |

Before inferring, then, from the weight of spleens as to the health or disease of the animal, it certainly may be demanded that the weight and age of the suspected animal also be given. There is abundant reason from the figures and statements of this report to believe that the spleens of cows in this climate are heavier and larger than those of Northern cattle. The average weight of all the spleens here reported was 1.98 pounds (almost exactly 2 pounds), and the reports are from all over the State. There is no part of the State where there is less reason to believe that disease exists among the cattle than in the vicinity of Forts McKavett or Mason; yet in Dr. Waters' report from there he says that "only three or four (spleens) weighed as low as 1½ lbs." In numerous cases where the spleen weighed 2½ lbs. and upwards, the observer has distinctly said that all the other organs seemed healthy, or that the animal appeared in good health. Our knowledge of the functions of the spleen is still quite imperfect. The numerous observations of Dr. Rauch, that where the spleen was of unusual size, the liver varied inversely—being unusually small—would at least give ground for suspicion that in these cases the functions of the two organs might be complementary.

Certainly the Darwinian can see no difficulties in the conclusion that a 2 lb. spleen may be a normal organ in Texas, and a 1½ lb. spleen a normal organ in Illinois.

Action of Light and Darkness, and of Different Colored Lights upon the Respiration of Lower Animals.—Jac. Moleschott and S. Fubini (*unters z. natural, d. menschen u. d. Thiere,*) have made some extended experiments which show the marked influence of light upon the excretion of carbonic acid gas. The facts obtained have an important bearing upon hygiene. The experiments were made with frogs, birds, rabbits, dogs, guinea-pigs, and water-rats. These animals were put in an apparatus in which the amount of $C.O._2$ exhaled per hour could be measured. The apparatus was so arranged, that the animals could be kept in the dark or in various colored lights. The authors give the following table showing the average variations in the excretion of carbonic acid gas in darkness and light, when the animal is blinded and when it is not:—

| Class of Animals. | Animal Uninjured. | | Animal Blinded. | |
|---|---|---|---|---|
| | In Darkness. | In Light. | In Darkness. | In Light. |
| Amphibious . . . . . . . . | 100 | 125 | 100 | 111 |
| Birds . . . . . . . . . . . | 100 | 134 | 100 | 127 |
| Mammals . . . . . . . . . | 100 | 140 | 100 | 112 |

This shows that light has an undoubted influence in promoting the excretion of $C.O._2$; also, that this influence is not due to any effect of the light upon the eyes. Other experiments showed that it is rather due to the effect of light on the skin.

The authors also found that light increased the activity of tissue respiration. Thus the muscles of a dog, in light, give off .408 grams (gr. vii.) of $C.O._2$; in the dark, only .231 grams (gr. iv.).

Different colored lights affect the $C.O._2$ excretion. Red light has a very weak action. But violet-blue and white lights have a powerful effect as respiratory stimulants.

The authors conclude that light has a very powerful effect upon the tissue changes of all the animals which they experimented upon (insects, amphibia, birds and animals).

CHLORAL V. STRYCHNIA.—The following facts may be of some interest to your readers. On May 25th, I found a pet fox terrier bitch suffering from severe symptoms of strychnia poisoning. There were well-marked risus sardonicus and opisthotonos, with much rigidity of the limbs. She was lying on her side, totally unable to get up, and the respiration was 90 to the minute. I gave her immediately a draught of bromide of potassium and chloral (thirty grains of each), but owing to the spasmodic movements of the head I failed to get it all down. I then gave her sixty grains of chloral (in crystals) on the tongue, and held her mouth until it was dissolved. She gradually went to sleep, and three hours after was able to walk, although with a good deal of weakness about the hind legs, and she has since gone on slowly to recover.—*London Lancet.*

CONTAGIOUS PYÆMIA OF RABBITS.—In some experiments on the inoculation of rabbits with the blood of animals dying from splenic fever, Professor Semmer, of Dorpat, has found that a disease is produced which is not anthrax nor ordinary septicæmia, but a form of pyæmia, which he believes to be new and specific. The animal inoculated with the blood of splenic fever died in two days with abscesses over the glutei at the place of inoculation, and ecchymosis and infarcts in internal organs. The blood was not decomposed, but presented an excess of white corpuscles, while the red corpuscles were beset with micrococci, which were found also in the serum of the blood and in the pus of the abscesses. A second rabbit, inoculated from the first, died in six days with very similar appearances, and micrococci were found also in the cells of the liver and in the tubules of the kidney. White points were also found in the liver, due to the accumulation there of colorless corpuscles. A long series of inoculations gave very similar results. An inoculated dog died on the third day with purulent conjunctivitis and a low form of pneumonia. The liver and kidneys were yellowish-brown, the liver cells granular. In the renal tubules there were detritus and fat-glanules, and colorless blood corpuscles in the glomeruli. At the place of inoculation pus had formed, which contained the same micrococci. Central experiments with ordinary pus yielded a negative result. The destruction of the pathological condition from ordinary contagious septicæmia is in the absence of any destruction of the blood corpuscles, the absence of transudation and inhibition, and of any rapid decomposition after death. The small micrococci appear to be the agents of its production. In many cases, there is no evidence of metastasis and infraction, which do not, therefore, appear to be an essential part of the morbid process.—*London Lancet.*

TRICHINA-LIKE PARASITES.—To microscopists who are endeavoring to ascertain the various habitats of trichinæ, a useful word of warning has been given by M. Mégnin in a paper read before the Societè de Biologie, in which he points out that many minute encysted worms are met with which are not trichinæ, although so clearly resembling them as to have deceived many observers. The supposed discovery of trichinæ in the rootlets of beetroot, proved to be a mistake by Virchow and Kuhn, is a striking instance of this sort. Lagenbeck discribed trichinæ in the intestines of earth-worms; but Kuhn showed that the parasite is quite distinct from the trichina sprialis. Merlan and Tigri thought they had found trichinæ in the lungs of sheep; but Deepech showed that these were merely the embryos of strongylus filaria. Cobbold has stated that the trichinæ is common in the hedgehog. Mégnin is convinced that this is an error, and that the worms described are merely the encysted larvæ of the spiroptera clausa. He showed preparations of an encysted nematoid worm, which might easily be mistaken for the trichina; but pointed out that the former differs in having a papilla at its mouth, and the anus is not terminal. Siebold describsd as a trichina a worm found in cysts in the peritoneum of the grey lizard and other creatures; but Mégnin asserts that this also is the larva of a spiroptera (S. abbreviata), the adult individuals of which are abundant in the intestines of the same animal. An encysted spiroptera still more strikingly resembling the trichina has been found in the muscles of the frog. Very similar, but larger, encysted worms of the same genus have also been discovered in the subcutaneous tissues of a bird (the manchetes pugnax).—*London Lancet.*

A New Mode of Treatment with Chloroform.—In an important paper relating to the use of anæsthetics communicated to the Paris Academy of Science by M. Paul Bert, the new French Minister of Public Instruction, experiments are described in which dogs, mice and sparrows were kept in chambers containing air along with various proportions of some anæsthetic. In a graduated series of such mixtures of increasing strength one is found just sufficient to cause insensibility, and proceeding higher a dose is reached which kills. The interval between these points (the anæsthetic dose and the fatal dose) M. Bert calls the working zone (*zone maniable*). He has sought to determine it for various agents—chloroform, ether, amylene, bromide of ethyl, chloride of ethyl—for the animals named, and has reached the singular result that in all these cases the fatal dose is precisely double the anæsthetic dose. Thus—*e. g.*, in the case of mice submitted to chloroform, six grammes of chloroform vapor in one hundred litres of air causes insensibility, and twelve grammes is fatal. When an animal is made to breathe, in the way indicated, a mixture about the middle of the working zone, it is very quickly anæsthetized, and remains perfectly quiet during the whole experiment (two hours in some cases), not requiring any attention or concern; and the contrast in this respect to the ordinary methods by compress, sponge, etc., is striking. In the latter case, indeed (M. Bert points out), a patient alternately breathes, according to the quantity of chloroform in the compress or its distance from mouth to nose, a mixture of air and chloroform, either below the active dose or within the working zone, or at or beyond the limit of safety; and a fatal result in the last instance is not always warded off by prompt removal of the compress. The working zone is often very narrow; in the case of chloroform, while eight grammes in 100 litres does not suffice to render a dog insensible, twenty grammes kills it. Ether is much less dangerous, for between the active and the fatal doses of it there is an interval of forty grammes. An anæsthetic acts, not by the quantity respired, but according to its proportion in the inspired air; hence the statements of surgeons as to how much chloroform they put on the compress have little value. M. Bert recommends the use of a mask, communicating by a tube, with a zinc reservoir holding 200 or 300 litres of the anæsthetic mixture. The pulse and the respiration need no attention. The most delicate matter would be the determination of the lower limiting dose. The author's experiments here give no guidance. The doses varied greatly for dog, mouse, and sparrow—always less for the mouse than the dog. They were always greater for the sparrow than for the mouse; and in the case of chloroform and amylene they were about equal for the sparrow and the dog. Among other facts, it is stated that the mixture alters very little in strength, except in the first instants. Experimenters have sometimes been mistaken as to the fatal proportion of chloroform in air, through using potash to absorb carbonic acid; this substance rapidly decomposes chloroform. Once more, the working zone for protozide of nitrogen is more extensive than for the substances specified; the ratio between the limiting doses being one to three.

